# Understanding the α-crystallin cell membrane conjunction

**Published:** 2011-10-26

**Authors:** Shih-Ping Su, Jason D. McArthur, Michael G. Friedrich, Roger J.W. Truscott, J. Andrew Aquilina

**Affiliations:** 1 School of Biological Sciences, University of Wollongong, Wollongong, NSW, Australia; 2 Save Sight Institute, University of Sydney, GPO Box 4337, Sydney, NSW, Australia

## Abstract

**Purpose:**

It is well established that levels of soluble α-crystallin in the lens cytoplasm fall steadily with age, accompanied by a corresponding increase in the amount of membrane-bound α-crystallin. Less well understood, is the mechanism driving this age-dependent membrane association. The aim of this study was to investigate the role of the membrane and its associated proteins and peptides in the binding of α-crystallin.

**Methods:**

Fiber cell membranes from human and bovine lenses were separated from soluble proteins by centrifugation. Membranes were stripped of associated proteins with successive aqueous, urea, and alkaline solutions. Protein constituents of the respective membrane isolates were examined by SDS–PAGE and western immunoblotting. Recombinant αA- and αB-crystallins were fluorescently-labeled with Alexa350^®^ dye and incubated with the membrane isolates and the binding capacity of membrane for α-crystallin was determined.

**Results:**

The binding capacity of human membranes was consistently higher than that of bovine membranes. Urea- and alkali-treated membranes from the nucleus had similar binding capacities for αA-crystallin, which were significantly higher than both cortical membrane extracts. αB-Crystallin also had a higher affinity for nuclear membrane. However, urea-treated nuclear membrane had three times the binding capacity for αB-crystallin as compared to the alkali-treated nuclear membrane. Modulation of the membrane-crystallin interaction was achieved by the inclusion of an NH_2_-terminal peptide of αB-crystallin in the assays, which significantly increased the binding. Remarkably, following extraction with alkali, full length αA- and αB-crystallins were found to remain associated with both bovine and human lens membranes.

**Conclusions:**

Fiber cell membrane isolated from the lens has an inherent capacity to bind α-crystallin. For αB-crystallin, this binding was found to be proportional to the level of extrinsic membrane proteins in cells isolated from the lens nucleus, indicating these proteins may play a role in the recruitment of αB-crystallin. No such relationship was evident for αA-crystallin in the nucleus, or for cortical membrane binding. Intrinsic lens peptides, which increase in abundance with age, may also function to modulate the interaction between soluble α-crystallin and the membrane. In addition, the tight association between α-crystallin and the lens membrane suggests that the protein may be an intrinsic component of the membrane structure.

## Introduction

The human lens grows continuously throughout life via the addition of new fiber cells on top of preexisting ones. This process leads to the formation of two lens regions referred to as the nucleus, consisting of fiber cells formed in utero, and an ever-expanding cortex which consists of fiber cells formed post-natally [1,2]. Lens fiber cells contain a high concentration of the structural proteins α-, β- and γ-crystallins, which contribute significantly to the transparent and refractive properties of the lens. α-Crystallin, which is composed of subunits αA and αB, functions additionally as a molecular chaperone [3,4]. As there is little to no protein turnover in mature lens fiber cells [5], the long-term transparency of the lens is dependent on the ability of α-crystallin to sequester unfolded proteins and maintain them in soluble chaperone-client complexes.

Lens proteins undergo a multitude of posttranslational modifications during aging, which over time contribute to the unfolding and denaturation of crystallins [6-12]. With age, the level of unfolded proteins eventually exceeds the chaperone capacity of α-crystallin in the lens, such that by 40 years, all free α-crystallin in the lens has been used [13-15]. Subsequently, the level of water-insoluble and high molecular weight crystallin aggregates in the lens increases significantly [16-19]. Large-scale binding of α-crystallin to lens membranes has also been documented in the lens with age [20-22], and this has been hypothesized to contribute to the development of nuclear cataract by occluding membrane pores, thus leading to the formation of a barrier to diffusion [22-24].

The association between crystallins and lens fiber cell membranes is believed to be an important process by which crystallin insolubilization occurs in the aging lens [22,25,26]. Chandrasekher and Cenedella [25] showed that essentially all water-insoluble crystallins in old human lenses are membrane-bound. α-Crystallin, in particular, has a strong affinity toward lens membrane [20,21,27-30], leading to the suggestion that the overall increase in the membrane binding of crystallins in the lens is due to other crystallins associating with membrane-bound α-crystallin [27]. However, little is currently known about the mechanism(s) by which α-crystallin associates with the cellular membrane, and how age-related changes to the membrane compositions may affect crystallin-binding.

In this study, we examined the protein constituents of bovine and human lens membranes after successive removal of extrinsic protein. We report the effects of residual membrane-associated proteins on the binding behavior of recombinant α-crystallin and the effect upon this binding of a known intrinsic lens peptide.

## Methods

### Isolation of bovine and human lens fiber cell membranes

A total of 142 human lenses, aged between 54 and 84 years, were obtained from donors at the Lions New South Wales Eye Bank (Sydney, Australia). Tissue was handled in accordance with the tenets of the Declaration of Helsinki. Fresh bovine lenses were obtained from a local abattoir. All lenses were stored at −80 °C before dissection.

Lenses were separated into cortical and nuclear regions using a 4.5 mm diameter trephine. A cold scalpel was used to remove 1 mm of tissue from each end of the resulting cylindrical center (nucleus). The cortical and nuclear tissues from human lenses and the cortical tissues from the bovine lenses were used in this study. Lens membranes were obtained using methods published by Russell et al. [31] with minor modifications. Briefly, lens tissues were homogenized in 7 times (w/w) of buffer A (50 mM Tris-HCl, 5 mM EDTA, 10 mM β-mercaptoethanol, 0.2% sodium azide, pH 7.4) and incubated at room temperature for 10 min. The homogenate was centrifuged at 15,000× g for 20 min at 4 °C, and the supernatant was discarded. The pellet was re-homogenized in buffer A and centrifuged at 15,000× g for another 20 min. The resulting pellet was washed with MilliQ™ water (Kilsyth, Vic Australia), centrifuged and re-homogenized in buffer B (50 mM Tris-HCl, 7 M urea, pH 7.4). The urea concentration in the homogenate was diluted to 3.5 M with water and the homogenate was centrifuged at 15,000× g for 20 min. This extraction procedure using buffer B was repeated and the resulting pellet was washed three times with water to give the urea-treated membrane. To obtain the alkali-treated membrane, a portion of the urea-treated membrane pellet was re-homogenized in solution containing 0.1 M NaOH, 1 mM β-mercaptoethanol. The homogenate was incubated on ice for 15 min and then centrifuged at 15,000× g for 20 min at 4 °C. The pellet was further washed with 5 mM phosphate (pH 8.0), followed by two washes with water.

Isolated membranes were re-suspended in binding buffer (137 mM NaCl, 2.7 mM KCl, 4.3 mM Na_2_HPO_4_, 1.4 mM K_2_HPO_4_, 5 mM MgCl_2_, pH 7.3). Quantification of membrane samples was achieved by weighing freeze-dried aliquots of the membrane suspension using an Excellence XS105 dual-range analytical balance (Mettler Toledo, Greifensee, Switzerland). The concentration of membrane tissue (mg/ml) in the suspension was calculated by averaging at least four separate aliquots. Light absorbance of membrane samples at different wavelengths (220–850 nm) was measured using a POLARstar Omega multifunction microplate reader (BMG Labtech, Offenburg, Germany).

### SDS–PAGE and western immunoblot analysis of bovine and human lens membranes

Proteins in the bovine and human lens membrane preparations were examined using SDS–PAGE and western immunoblots. Samples were homogenized in SDS–PAGE loading buffer (60 mM Tris-HCl, 1% SDS, 1% β-mercaptoethanol, 10% glycerol, 0.01% bromophenol blue, pH 6.8) and incubated at room temperature for 2 h before loading onto 12% SDS–PAGE gels. Heating of the sample was avoided as elevated temperatures can damage the structure of intrinsic membrane proteins [31]. All SDS–PAGE gels were stained with Coomassie Blue R (Sigma-Aldrich, St. Louis, MO). For western immunoblot analysis, antibodies which specifically recognize human and bovine filensin, phakinin, αA- and αB-crystallins were used and were diluted to their working concentrations using PBS/T (137 mM NaCl, 2.7 mM KCl, 12.5 mM Na_2_HPO_4_, 1.8 mM KH_2_PO_4_, 0.05% (v/v) Tween-20, pH 7.4) containing 0.5% (w/v) skim milk powder. Proteins separated by SDS–PAGE were transferred onto polyvinylidene fluoride membranes at 30 V overnight at 4 °C using the Mini Trans-Blot system (Bio-Rad, Hercules, CA). Membranes were blocked for 1 h at room temperature in PBS/T containing 5% (w/v) skim milk powder. After three 10 min washes with PBS/T, the membranes were incubated with either a 1:1,000 dilution of polyclonal anti-αA-crystallin antibody (ab69552; Abcam, Cambridge, MA), a 1:1,000 dilution of polyclonal anti-αB-crystallin antibody (ab13497; Abcam), a 1:1,500 dilution of monoclonal anti-filensin antibody (F1043; Sigma-Aldrich), or a 1:5,000 dilution of monoclonal anti-vimentin antibody (V6389; Sigma-Aldrich) for 90 min at room temperature. Following another three 10 min washes with PBS/T, the membranes were incubated for 1 h with a 1:1,500 dilution of anti-rabbit immunoglobulin G (IgG) horseradish peroxidase (HRP) conjugate (65–6120; Invitrogen, Carlsbad, CA) for anti-αA-crystallin and anti-αB-crystallin blots, or a 1:2,500 dilution of goat anti-mouse IgG HRP conjugate (SAB-100J; Stressgen, Ann Arbor, MI) for anti-filensin and anti-vimentin blots. Excess secondary antibodies were removed by three 10 min washes with PBS/T, and the blots were developed in 100 mM Tris-HCl (pH 7.6) containing 1.4 mM diaminobenzidine and 0.015% (v/v) hydrogen peroxide.

### Quantification of phospholipids

Phospholipids were quantified by mass spectrometry as described previously [32]. Briefly, lenses were frozen under liquid nitrogen and ground using a mortar and pestle. Chloroform:methanol (2:1 v/v) containing 0.01% butylated hydroxytoluene was added to the lens material at a ratio of 20:1 solvent to tissue (v/w). After rotating overnight, total lipids were extracted, and quantified using a Waters QuattroMicro™ (Waters, Manchester, U.K.) equipped with a z-spray electrospray ion source and controlled by Micromass Masslynx version 4.0 software. To identify each phospholipid, a combination of precursor and neutral loss scans were used to detect the head-group of each phospholipid class [33]. Each phospholipid class was quantified by comparing their peak areas with the appropriate internal standard after correction for isotope contributions [32]. The percentage of each phospholipid was than determined by its contribution to the total phospholipid.

### Overexpression and purification of recombinant human αA- and αB-crystallins

The pET23d(+) plasmid, containing human wild-type αA-crystallin gene, was a gift from Prof. J. M. Petrash (Washington University, St. Louis, MO). The pET24d(+) plasmid (Novagen, Madison, WI), containing human wild-type αB-crystallin gene, was a gift from Prof. W. de Jong (University of Nijmegen, Nijmegen, The Netherlands). Plasmids were transformed into electro-competent *Escherichia coli* BL21 cells by electroporation using a GenePulser II system (Bio-Rad). LB stocks (900 ml; 1% tryptone, 0.5% yeast extract, 1% NaCl) that contained ampicillin (100 µg/ml) or kanamycin (50 µg/ml) were inoculated with 100 ml of overnight liquid cultures of BL21/pET23d-αA or BL21/pET24d-αB cells, respectively, and grown at 37 °C under vigorous shaking. The culture was induced with 10 ml of 50 mM isopropyl β-D-1-thiogalactopyranoside after reaching an optical density of ~0.5 at 600 nm, and further incubated for 3 h at 37 °C. Cells were harvested by centrifugation, and the expressed proteins were isolated using methods by Horwitz et al. [34]. Briefly, frozen cell pellets were resuspended in ice-cold lysis buffer (50 mM Tris-HCl, 100 mM NaCl pH 8.0) containing phenylmethanesulfonylfluoride (130 µM) and 800 µg/ml lysozyme.  After incubation on ice for 20 min, deoxycholic acid (4 mg per g of starting pellet) was added followed by vigorous shaking at 37 °C.  Cell disruption was completed using a French Press Cell Disruptor as per manufacturer’s instructions (ThermoFisher Scientific, Scoresby Vic Australia).  Cell debris was removed from lysates by centrifugation at 17,000× g for 15 min at 4 °C. Recombinant human αA and αB-crystallin were purified from the cell lysates using anion-exchange chromatography (HiPrep 16/10 DEAE FF column; GE Healthcare, Little Chalfont, UK), followed by size-exclusion chromatography (HiPrep 26/60 Sephacryl S-300 column; Amersham Biosciences, Uppsala, Sweden).

### Conjugation of Alexa350^®^ to α-crystallin subunits

Alexa350^®^ fluorescent tag (410 Da) was conjugated to recombinant αA- and αB-crystallins using an Alexa Fluor^®^ 350 Protein Labeling Kit (A10170, Molecular Probes, Carlsbad, CA), in accordance with the manufacturer’s instructions. Briefly, recombinant proteins in PBS (2 mg/ml) were mixed with 1 M sodium bicarbonate (10:1 v/v). The reactive dye was added to the protein solution and left for 1 h at room temperature.  Unincorporated dye was removed by size exclusion chromatography using BioGel P-30 Fine resin supplied with the kit. Purified Alexa350^®^-conjugated αA- and αB-crystallins were measured using absorbance readings at 280 nm and 346 nm (A_280_ and A_346_) on a UVmini-1240 UV-VIS spectrophotometer (Shimadzu, Kyoto, Japan). The protein concentration and the degree of binding were determined using the following equations:

$$[\text{protein}]\;\frac{\text{A}280\text{ - }(\text{A}346\;\times \;0.19)}{\text{εprotein}}\;\times \text{ dilution (Equation 1)}$$

$$\text{mol of dye/mol of subunit = }\frac{\text{A}346\text{ - dilution}}{\text{19000 × [protein]}}\text{ (Equation 2)}$$

Where 0.19 is a correction factor to account for absorption of Alexa350^®^ at 280 nm, 19000 is the molar extinction coefficient for Alexa350^®^ and ε_protein_ is the molar extinction coefficient for α-crystallin.

### Membrane binding measurements

Alexa350^®^-conjugated αA- and αB-crystallins were incubated with bovine and human lens membranes in binding buffer at 37 °C for 6 h, based on protocols established previously [30]. Control assays were set up by replacing Alexa350^®^-α-crystallin subunits with unlabeled α-crystallin subunits. All assays were performed in triplicate. After incubation, samples were centrifuged at 14,000× g for 30 min at 4 °C. The pellets were re-suspended in 30 μl of binding buffer and centrifuged again at 14,000× g for 30 min at 4 °C. The supernatants from both centrifugations were pooled together. The resulting pellets, which contained the membrane, and bound Alexa350^®^-α-crystallin subunits, were re-suspended in a volume of binding buffer equivalent to that of the combined supernatants. Both the supernatants and the re-suspended pellets were transferred to 96-well polypropylene black microplates (Greiner Bio-One, Frickenhausen, Germany), and the fluorescence reading (*F*) of the samples was measured using a FLUOstar Optima multifunction microplate reader (BMG Labtech). The excitation and emission filters were set at 320–340 and 430–450 nm respectively. The degree of binding (*X*) was determined by the following formula, 

where background fluorescence readings were obtained from control assays (using unlabeled α-crystallin):

$$\bar{X}\;=\;\frac{F\text{pellet - }F\text{pellet background}}{(F\text{pellet - }F\text{pellet background) + (}F\text{supernatant - }F\text{supernatant background)}}\text{ (Equation 3)}$$

The amount of α-crystallin subunits bound to the membranes was calculated by multiplying the total mass of Alexa350^®^-α-crystallin subunits used in the assay by the degree of binding (*X*). 

### Effect of endogenous crystallin peptides on the membrane binding of α-crystallin

Peptide αB_1–18_ (Ac-MDIAIHHPWIRRPFFPFH) was purchased from Biomatik (Cambridge, Ontario, Canada) with sample purity ≥95%. Peptide γS_167–178_ (SPAVQSFRRIVE) was purchased from Peptide 2.0 (Chantilly, VA) with sample purity of ≥91%. Peptides were solubilized in binding buffer to give a 4 g/l stock solution. Peptides were drawn from the stock solution and incubated with human lens membranes (80 μg) for 30 min at 37 °C. These were then incubated for 6 h at 37 °C in the presence of 24 μg of Alexa350^®^-conjugated αA- or αB-crystallin, or 24 μg of unlabeled αA- or αB-crystallin as negative controls. Binding assays were performed as described previously. Peptide interaction data were analyzed using unpaired two-sample *t*-test.

## Results

### Analysis of proteins associated with lens fiber cell membranes

#### Bovine lens membranes

**Figure 1:**
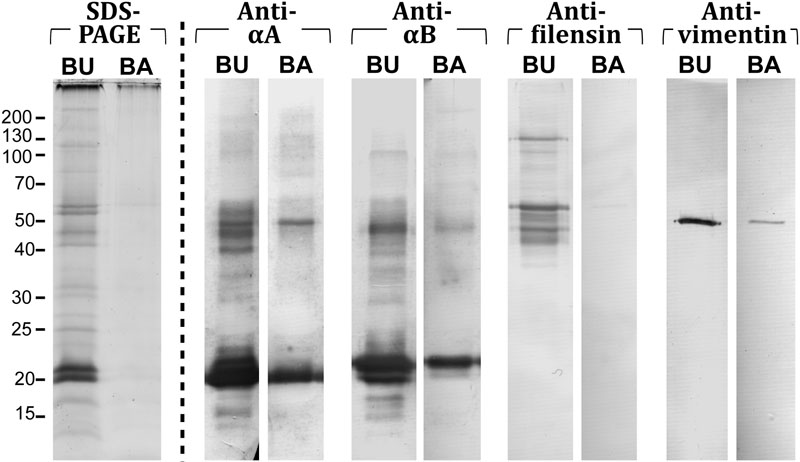
SDS–PAGE and western immunoblot analysis of bovine lens membranes. The water-insoluble cell membrane pellets from bovine lens homogenates washed with 7 M urea (BU) and with 0.1 M NaOH (BA). Samples were analyzed by SDS–PAGE (100 μg of membrane per lane) and western immunoblotting (20 μg of membrane per lane). Blots were probed using anti-αA-crystallin, anti-αB-crystallin, anti-filensin and anti-vimentin antibodies, as indicated. Molecular weight markers are on the left of the figure.

SDS–PAGE analysis of the urea-treated bovine (BU) membrane (Figure 1) showed that multiple proteins, ranging from 10 kDa to above 200 kDa, remain bound to the bovine lens membrane after the combined extractions of Tris- and urea-containing buffers. α-Crystallin subunits A and B (~20 kDa) were the predominant species detected, as shown by western immunoblots (Figure 1). In contrast, the alkali-treated bovine (BA) lens membrane extract contained no protein that could be detected by Coomassie staining.

Western immunoblots (Figure 1) revealed that both αA- and αB-crystallins (both ~20 kDa) were present in the BU and BA lens membranes, with a significant amount of higher molecular weight immuno-reactive protein also detected in the BU. Following further treatment of the membranes with alkali, α-crystallin subunits and a ~50 kDa αA species of protein remained (Figure 1). Of the two major intermediate filament proteins of the lens fiber cell cytoskeleton, intact filensin (115 kDa) and multiple cleavage products of filensin were detected only in the BU membrane, while vimentin (54 kDa) was present in both bovine lens membrane preparations (Figure 1).

#### Human lens membranes

**Figure 2:**
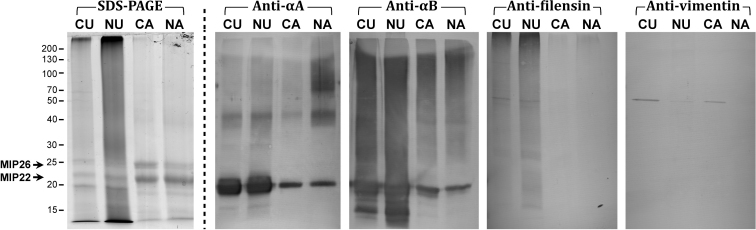
SDS–PAGE and western immunoblot analysis of human lens membranes. Isolated cortical and nuclear lens membranes were extracted with 7 M urea in Tris buffer (CU – cortex urea; NU – nucleus urea) and with 0.1 M NaOH (CA – cortex alkali; NA – nucleus alkali). Samples were analyzed by SDS–PAGE (100 μg of membrane per lane) and with western immunoblotting (20 μg of membrane per lane). Blots were probed using anti-αA-crystallin, anti-αB-crystallin, anti-filensin and anti-vimentin antibodies, as indicated. Molecular weight markers are on the left of the figure.

SDS–PAGE analysis (Figure 2) revealed human lens membrane to be very different from bovine lens membrane. Three proteins of ~20 kDa, 22 kDa and 26 kDa, which correspond to αA/αB-crystallin, major intrinsic protein (MIP) 22 and MIP26 respectively, were present in all human lens membrane preparations (Figure 2). The urea-treated membrane preparations, particularly urea-treated nucleus (NU) membrane, showed noticeable smearing down the length of the gel. In addition, a low molecular weight (LMW) band which migrated with the dye-front was observed, which was particularly intense in the urea extracts. The presence of this band indicates that significant amounts of LMW peptides, likely derived from crystallins [35-37], remain tightly associated with the lens membranes after extraction with urea.

Western immunoblots detected intact αA- and αB-crystallins (~20 kDa) in all human lens membrane preparations, even after the membranes had been stripped of the majority of extrinsic proteins with strong alkali solution (Figure 2). A diffuse band at ~43 kDa was also present in each of the αA-crystallin blots, with additional smearing between 70 kDa and 130 kDa, being particularly noticeable in the alkali-treated nuclear (NA) preparation. On the anti-αB-crystallin blots, extensive smearing was observed in the lanes of the urea-treated membranes, some of which may be attributed to non-specific immunoreactivity as described above. Interestingly, a variety of smaller αB-crystallin products less than 20 kDa remained bound to the lens membrane after urea-treatment (Figure 2). Most of the anti-αB reactive proteins were removed upon treatment with 0.1 M NaOH, except for some intense smearing from 90 to 150 kDa. Smearing was also observed on the anti-filensin blots of the urea-treated human lens membrane, while no filensin was detected in the alkali-treated membranes (Figure 2). Vimentin was only detected in the cortical human lens membrane preparations (Figure 2).

### Binding of α-crystallin subunits to lens membranes

#### Purification of Alexa350^®^-α-crystallin subunits

Recombinant human αA- and αB-crystallins were overexpressed in *E. coli* and conjugated to Alexa350^®^. On average, 0.56±0.07 mol of Alexa350^®^ was attached per mol of αA-crystallin, and 0.44±0.36 mol of Alexa350^®^ per mol of αB-crystallin.

#### Bovine lens membrane binding

**Figure 3:**
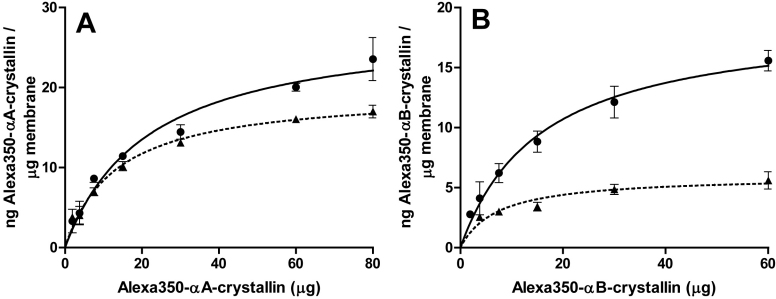
Binding of α-crystallin to bovine lens membrane preparations. Binding curves depicting the amount of bound Alexa350^®^-conjugated αA- (A) and αB-crystallin (B) per μg of urea-treated (●) or alkali-treated (▲) bovine lens membrane as a function of the free α-crystallin concentration. Bovine lens membranes (200 μg) were incubated with a mass range of Alexa350^®^-conjugated αA- and αB-crystallins, for 6 h at 37 °C. Results are the mean±SEM (n=3).

To compare the binding of α-crystallins to bovine and human lens membranes, we pre-labeled recombinant αA- and αB-crystallins with Alexa350^®^, whose excitation and emission characteristics are spectrally distinct from aromatic amino acids. Binding assays using BU and BA membranes displayed saturable binding behavior (Figure 3), which allowed us to calculate the membrane binding capacity from the asymptote of each curve (Table 1). The binding capacities for αA-crystallin were 28.1±2.4 ng αA/μg BU membrane, and 19.5±0.8 ng αA/μg BA membrane. The binding capacities for αB-crystallin were lower, being 19.1±1.1 ng αB/μg BU membrane and 6.0±0.6 ng αB/μg BA membrane.

**Table 1:** Binding capacity of bovine and human lens membranes for Alexa350^®^-α-crystallin subunits.

**Membrane samples**	**αA binding capacity (ng/μg membrane)**	**αB binding capacity (ng/μg membrane)**
BU	28.1±2.4	19.1±1.1
BA	19.5±0.8	6.0±0.6
CU	31.5±2.0	54.3±4.2
CA	33.4±2.8	31.6±1.8
NU	95.4±4.2	208.0±13.1
NA	78.5±2.0	67.0±2.4

Abbreviations used: BU – urea-treated bovine membrane; BA – alkali-treated bovine membrane; CU – urea-treated human cortical membrane; CA – alkali-treated human cortical membrane; NU – urea-treated human nuclear membrane; NA – alkali-treated human nuclear membrane.

#### Human lens membrane binding

**Figure 4:**
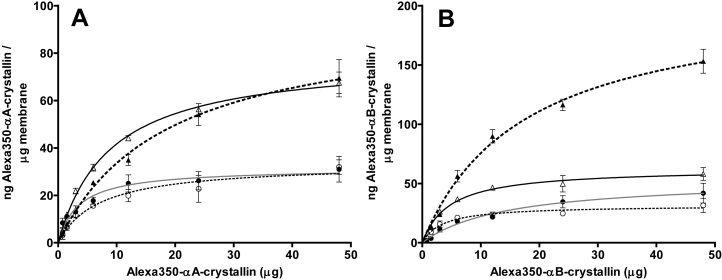
Binding of α-crystallin to human lens membrane preparations. Binding curves depicting the amount of bound Alexa350^®^-conjugated αA- (**A**) and αB-crystallin (**B**) per μg of urea-treated cortical (●) and nuclear (▲) human lens membrane, or alkali-treated cortical (○) and nuclear (△) human lens membrane as a function of the free α-crystallin concentration. Human lens membranes (80 μg) were incubated with a mass range of Alexa350^®^-conjugated αA- and αB-crystallins, for 6 h at 37 °C. Results are the mean±SEM (n=3).

All of the assays using human lens membranes displayed saturable binding of the Alexa350-α-crystallins (Figure 4), and the binding capacity was greater, on the whole, than bovine lens membranes (Table 1). Comparing regions, a matched treatment analysis revealed that nuclear membrane had significantly greater binding capacity for the α-crystallins than cortical membrane (p<0.05). Matched region analysis indicated the difference in binding due to solvent treatment is less significant (p>0.1). For example, αA-crystallin was observed to bind NU and NA membrane with less than 20% difference in capacity, while CU and CA displayed almost identical αA-crystallin binding capacity. αB-Crystallin, however, exhibited significantly greater binding to the NU membrane than the NA membrane (208.0±13.1 ng/μg compared with 67.0±2.4 ng/μg). This result strongly suggests that proteins removed from the nuclear membrane by the alkali treatment have a much higher affinity for αB-crystallin than the extrinsic proteins of the cortex membrane.

Membrane lipid analysis revealed no significant alteration in the ratios of the eight major classes (dihydrosphingomyelin, sphingomyelin, phosphatidylcholine, phosphatidylserine, ceramide, dihydroceramide, phosphatidylethanolamine, and lysophosphatidylethanolamine) between the human membrane preparations used in these binding assays (Figure 1A, Appendix 1), demonstrating that membrane binding of α-crystallin was not influenced by changes to the proportion of individual membrane lipids. Human membrane preparations displayed no significant absorbance between 380 and 500 nm (Figure 2A, Appendix 2), indicating there would be minimal interference with the fluorescent signal emitted by the Alexa350^®^ dye.

### Effect of crystallin peptides on the membrane binding of α-crystallin subunits

**Figure 5:**
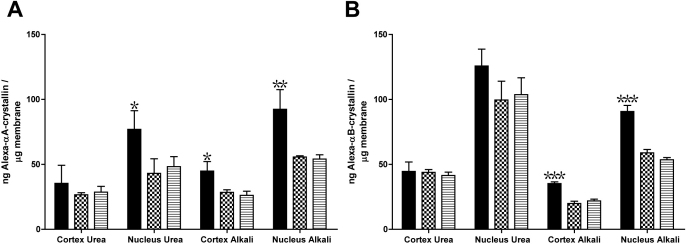
Effect of crystallin peptides on the binding of α-crystallin to human lens membranes. Graphs showing the amount (ng) of Alexa350^®^-conjugated αA- (**A**) or αB-crystallin (**B**) bound per μg of human lens membrane in the presence of crystallin peptides. Human lens membranes (80 μg) were mixed with either 48 μg of peptide αB_1–18_ (solid bars), 48 μg of peptide γS_167–178_ (dotted bars), or with binding buffer only (horizontal line bars), for 30 min at 37 °C. Alexa350^®^-conjugated αA- or αB-crystallin (24 μg) was then added and the mixtures were incubated for 6 h at 37 °C. Results are the mean±SEM (n=3). *p<0.05, **p<0.01, ***p<0.001 compared with assays performed with binding buffer only (horizontal line bars).

In a recent study, we reported that crystallin peptides αB_1–18_ and γS_167–178_ were present in human lenses across a broad age-range [36]. Santhoshkumar et al. [35] reported that peptide αB_1–18_ possessed anti-chaperone activity against α-crystallin, while peptide γS_167–178_ had little effect. Peptide αB_1–18_ was found to significantly increase the amount of membrane-bound α-crystallin subunits in all but the CU membranes (Figure 5). In the case of αA-crystallin, the addition of peptide αB_1–18_ increased the binding by 59% (NU membrane), 72% (CA membrane) and 72% (NA membrane). Similar results were observed in binding assays using αB-crystallin, with the amount of membrane-bound αB-crystallin increasing by 61% (CA membrane) and 69% (NA membrane). However, peptide αB_1–18_ did not give rise to a significant increase in binding of αB-crystallin to the urea-treated membranes (Figure 5B), possibly due to the amount of endogenous αB-crystallin that is present on this membrane preparation (Figure 2). The addition of peptide γS_167–178_ had a negligible effect on the binding of both αA- and αB-crystallins to all human lens membrane preparations (Figure 5).

## Discussion

There is growing evidence that association with fiber cell membranes is an important process by which crystallins become water-insoluble in the lens [22,25,30]. This association may also contribute to the age-dependent loss of free α-crystallin [13-15]. In addition, large-scale binding of crystallin aggregates after middle age [22,23] is implicated in the development of the lens barrier – a significant event in the human lens that is associated with subsequent cataractogenesis [24,38,39]. Building upon previous studies, where the membrane binding of α-crystallin was characterized using non-human lens tissues [26,30,40-44], here we determined the binding properties of α-crystallin to membranes isolated from both bovine and human lens tissues.

αA- and αB-crystallins were the dominant membrane-bound proteins in the bovine lens after extraction with urea, whereas the human membrane also contained significant amounts of aquaporin 0 (AQP0, [MIP26, MIP22]), small peptides and, particularly in the nucleus, a complex mixture of cytoskeletal and crystallin proteins. Full-length αA- and αB-crystallins remained tightly associated with both the bovine and human lens membranes after extraction with 7 M urea, and remarkably, even after the alkali-treatment. The presence of α-crystallin after such vigorous treatment indicates that it is very tightly associated with, or even partially embedded in the fiber cell membrane. To our knowledge, this is the first evidence which suggests that α-crystallins may be an intrinsic component of the lens fiber cell membrane. Given the important role that α-crystallin has on the structural assembly of the beaded filaments [45,46], the incorporation of α-crystallin into the fiber cell membrane structure may have functional significance regarding the stability and order of the lipid bilayer. Furthermore, fatty acid acylation, which confers membrane anchoring properties to proteins, and has been reported for lens AQP0 [47], may also contribute to the tight association between α-crystallin and the lens membrane, if α-crystallin subunits are acylated in a similar manner.

We observed that the level of αB-, but not αA-crystallin, binding to the bovine and human membranes was influenced by the extent to which the membrane had been stripped of extrinsic proteins. This may be due to the fact that αB-crystallin is a more potent chaperone at physiologic temperatures (37–40 °C) compared to αA-crystallin [48], and that membrane-bound proteins represent potential chaperone targets. As the alkali-treated membranes were largely stripped of this membrane-bound protein, the interacting targets for αB-crystallin decreased significantly, thus lower binding capacity was observed. These results suggest that the mechanisms by which αA- and αB-crystallins associate with the cellular membrane are different; αA-crystallin may interact exclusively with membrane phospholipids, thereby being unaffected by the presence of extrinsic proteins on the membrane, whereas these proteins may act as conduits for αB-crystallin to bind to the membrane.

Membrane binding of both αA- and αB-crystallins was significantly increased in the presence of peptide αB_1–18_. It has previously been reported that when mixed with wild-type αB-crystallin, peptide αB_1–18_ increased the oligomeric size of the protein [49]. Thus, our results may reflect the fact that larger α-crystallin oligomers containing greater numbers of α-crystallin subunits are binding to the membrane. Considering that the emergence of this peptide in the lens nucleus [36] corresponds to the time when large-scale binding of α-crystallin to lens membrane is observed [22], this and other endogenous peptides [35,36] may play a role in this process.

Previous studies into membrane binding of α-crystallin have produced conflicting outcomes. For example, Ifeanyi et al. [42] found that the association of α-crystallin to membranes was mainly due to the αA subunit. In contrast, Cobb and Petrash [30] suggested that αA and αB subunits have similar binding capacities to lens membranes. Typically, membrane binding studies were performed using non-human lens membranes. As the membrane binding of α-crystallin is affected by the presence of membrane-bound proteins, and endogenous peptides, the use of non-human tissue does not appear to represent a useful model for the aging human lens.

Recent studies have also shown that the lipid composition of human lens membrane differs considerably from that of other mammalian animals [32]. The levels of dihydrosphingomyelin and cholesterol are much higher in human lenses [32], and these hydrophobic components [50] could mimic the exposed hydrophobic residues on substrate proteins, with which α-crystallin is known to interact [51,52]. Another characteristic of the human lens is that its phospholipids undergo major changes with age. The levels of glycerophospholipids in the nucleus of the lens decline steadily, such that by age 40, their content is negligible [53]. In contrast, the levels of ceramides and dihydroceramides in the lens nucleus increase approximately 100 fold during middle-age [53]. It is therefore possible that age-related changes to membrane phospholipids are influential in the binding of α-crystallin and the persistence of this versatile small heat-shock protein may also be an important component of the fiber cell membrane itself.

## Appendix 1. Relative ratio of phospholipids in human lens membrane preparations.

Shown are the relative quantities of phospholipids (DHSM: dihydrosphingomyelin, SM: sphingomyelin, PC: phosphatidylcholine, PS: phosphatidylserine, PE: phosphatidylethanolamine, Cer: ceramide, DHC: dihydroceramide and PE lyso: lysophosphatidylethanolamine) present in the cortical urea (CU, red bars), cortical alkali (CA, green bars), nuclear urea (NU, yellow bars) and nuclear alkali (NA, blue bars) human lens membranes. To access the data, click or select the words “Appendix 1.” This will initiate the download of a compressed (pdf) archive that contains the file.

## Appendix 2. Relative absorbance of the human lens membrane.

Graph showing the relative absorbance of the urea-treated cortical (dark blue line) and nuclear (red line) membranes, and the alkali-treated cortical (green line) and nuclear (violet line) membranes between 380-500 nm. Binding buffer (light blue line) was used as negative control. To access the data, click or select the words “Appendix 2.” This will initiate the download of a compressed (pdf) archive that contains the file.
